# Children’s Self-Reported Reasons for Accepting and Rejecting Foods

**DOI:** 10.3390/nu11102455

**Published:** 2019-10-14

**Authors:** Julia Sick, Rikke Højer, Annemarie Olsen

**Affiliations:** 1 Department of Agriculture, Food, Environment and Forestry, University of Florence, Via Donizetti 6, 50144 Florence, Italy; julia.sick@unifi.it; 2 Department of Food Science, Faculty of Science, Section for Design and Consumer Behaviour, University of Copenhagen, Rolighedsvej 26, 1958 Frederiksberg C, Denmark; rikke.h@food.ku.dk; 3 University College Absalon, Center for Nutrition & Rehabilitation, Nutrition & Health, Slagelsevej 72, 4180 Sorø, Denmark

**Keywords:** food choice, acceptance, rejection, children, eating behavior, food, CATA

## Abstract

Children’s eating behavior does not necessarily align with dietary recommendations, and there is a need for better understanding the factors underlying their food choices. The aim of this study was to investigate children’s self-reported reasons for accepting and rejecting foods. A questionnaire was developed with reasons based on prior research and in-depth interviews. A set of various food stimuli covering different types was evaluated by 106 girls and 99 boys aged 10–13 years by checking all reasons that apply (CATA) for either accepting or rejecting them. Results showed gender differences among reasons for both food acceptance and rejection, but also in liking and willingness to re-taste the stimuli. The most common reason for food acceptance was good taste in boys and curiosity in girls; for food rejection they were bad taste, bad smell and dislike of appearance in boys and bad taste, bad smell, dislike of appearance and texture in girls. Overall, boys liked the food stimuli more than girls and were more willing to re-taste them. Future research should focus more on the role of sensory properties in both acceptance and rejection, and the potential of children’s curiosity as a driver in tasting foods should be further explored.

## 1. Introduction

Dietary variety has been linked to nutritional status and is therefore important for health [1]. However, a recent Danish cross-sectional study showed that school children consumed insufficient amounts of fruits and vegetables, fish and dietary fiber, but an excess consumption of red meat, saturated fats and sugars [2]. Insufficient dietary variety is often linked to picky eating and food neophobia [3], but to promote healthy food habits, more knowledge is required about factors that determine food choice. Fallon and coworkers have proposed four main reasons for rejection including distaste, danger, disgust and inappropriateness and four main reasons for acceptance of foods including good taste, benefit, appropriateness and transvalue [4,5], whereof some of the factors already appear during childhood [6].

Koivisto and Sjödén (1996) investigated reasons for liking and disliking foods in 2–17-year-old children and discovered that distaste was the main reason for disliking and good taste the main reason for liking specific foods. Other important reasons children stated for dislike were “texture” and “negative consequences” or they were not able to indicate the reason and responded with “don’t know”. The importance of taste and texture has also been highlighted in other studies [7,8,9], although a review on children’s exposure to healthy foods concluded that liking and familiarity are the most important factors determining children’s food choice. The importance of sensory properties was highlighted as one of the most influential factors determining eating behavior [9,10,11,12,13] and within these, good taste, smell and appearance [7,14,15] and texture [15] were shown as the basic requirements of food consumption and indicators of whether a food is eaten or not.

Currently, there is some inconsistency to which factors mostly determine children’s acceptance or rejection of food and there is also a lack of knowledge about children’s self-reported reasons. Most research in this field has examined children’s reasons indirectly, where factors influencing food choice were described by academic professionals or by asking parents [8,16,17]. In fact, research conducted by asking children directly seems to be limited, although recently children have been recognized as an important consumer group that are able to conduct consumer tests by describing different food products [18,19,20]. Asking children directly through questionnaires could give a better understanding to why they accept some foods while rejecting others. Especially, as children grow older, they gain more autonomy when it comes to choosing food and following take their own decisions about what foods they want or don’t want to eat. Additionally, the direct exposure to food can evoke various reactions (i.e., physical or physiological) and stimulate the senses from person to person differently leading to individual differences in taste perception [21]. Hence, a parent or caregiver usually responds very subjectively and might not reflect the child’s own food experience very well. Therefore, it is important to examine children’s direct responses, although there might be indirect factors (i.e., biological, contextual, environmental, experiential, emotional) they are not aware of when accepting or rejecting food [20,22,23]. Both direct and indirect factors are needed to better understand children’s food choices. In addition, many studies focus on the rejection of food [16,22,24,25], but also knowledge about food acceptance is essential for a more complete comprehension of food choice [26]. There are several interesting health implications of such research, as such knowledge could be used by parents, health care practitioners, and school canteens alike to facilitate and support healthy eating behavior and encourage children to eat a wide range of foods.

Moreover, there seem to be differences between genders in relation to children’s selection of food. It was detected that girls liked vegetables [10,11,27,28] and fruits [27,28] more than boys and that boys liked fatty and sugary foods, eggs [27], meat [12,27], fish and poultry [12] more than girls. Furthermore, it was demonstrated that males and females differed in comfort food preferences, where females showed a higher preference for snack foods, whereas males preferred foods that were described as hearty, warm and comfort foods related to meals [13]. However, this is in contrast with some research that could not show any or only minor gender differences in food preferences for specific food groups [10,25,29]. Women were shown to be more disgust sensitive than men [25], but when investigating food-related personality traits in children, boys were shown to be more food neophobic compared to girls [30,31]. Another study revealed gender-specific differences concerning food packaging influences. Children were more likely to choose gender-consistent packaging of a snack, even if a snack was offered that was tastier and did not have gender-consistent packaging [32]. As gender differences have been shown in relation to different aspects of food choice [27], it is also crucial to investigate gender differences in reasons for accepting and rejecting food. For instance, food provided in e.g., school canteens, is usually the same for both genders, but if different parameters drive acceptance and rejection among boys and girls, this is important to know in order to implement approaches—gender-neutral or gender-specific—that will contribute to increase acceptance of healthy foods among both genders. 

Based on previous research we hypothesized that children base their food choices mainly on sensory attributes and that there are gender-specific differences in the reasons given for accepting or rejecting specific foods. Accordingly, the aim of the present study was to examine children’s self-reported reasons for accepting and rejecting food and to investigate possible gender differences related to these.

## 2. Materials and Methods

### 2.1. Participants

The study comprised 106 girls and 99 boys aged 10–13 years and the average age was 11.0 ± 0.1 (mean ± standard error of the mean (SEM)) for both genders. Recruitment was done by sending invitation letters to public schools in Copenhagen, Denmark, whereof 10 school classes from five public secondary schools agreed to participate. Parents were asked to give written consent and to state if their child had any food allergies. It was voluntary for the children to participate. The procedures were in accordance with the Helsinki declaration. In Denmark this type of research does not require formal ethical approval, and it is thus not possible to obtain it.

Previously, a pilot study was run to test the study procedure with 15 girls and 6 boys both aged 9–10 years attending a public school in Copenhagen that followed the same participation criteria as mentioned above.

### 2.2. Selection of Stimuli

A broad spectrum of food groups was included, as previously conducted studies showed that boys and girls have different preferences for various food groups [12,27] and consequently would facilitate a range of reasons for liking or disliking these. One-on-one interviews (60 min) were conducted with four children aged 9–10 years, which were presented with different food stimuli presented as food images to explore, to assess which foods would elicit the highest number and diversity of reasons for acceptance and/or rejection. Each child was shown 28 food images including a set of fruit, vegetable, meat-based, fish-based and dairy products. Consequently, 14 food images were selected from the interviews and tested as real food stimuli in a piloted tasting session. All products were required to be easily available in local supermarkets, have affordable prices and be suitable for handling and serving whole classes simultaneously. Additionally, potentially allergenic foods to children were considered [33]. As most of the stimuli were accepted in the pilot study, some of the stimuli were excluded or replaced with stimuli that are usually disliked and rejected by children to increase the number of reasons for rejected stimuli [24]. It was also observed that with the serving of the 10th food stimulus, children started to lose concentration. Consequently, the stimuli used in the main study included nine food stimuli: pumpkin (pickled and cubed; Samsø Syltefabrik), kale (raw and sliced; Coop), seaweed (dried dulse; *Palmaria palmata*; Dietz Seaweed), physalis (served as whole fruit; Coop), caviar (lumpfish roe from *Cyclopterus lumpus*; Vores^®^), herring (pickled and sliced in small pieces; Princip), anchovy (pickled and sliced in pieces; Lykkeberg), blue cheese (cut in cubes; Castello^®^) and deer salami (sliced in quarters; Deli del Toro, Copenhagen). Prior to study execution, the stimuli were cut into equal bite-sized pieces that were distributed and presented in tasting cups (30 mL).

### 2.3. Selection of Reasons for Acceptance and Rejection

Another part of the one-on-one interviews on the selection of food stimuli was also to interview the same children about their reasons for accepting or rejecting food based on their most liked and most disliked foods. The aim was to open a discussion about children’s most liked and disliked food stimuli and their reasons for them. First, children were asked to draw these foods on a provided sheet to make them feel at ease. All interviews were audio recorded and transcribed verbatim. The children reported 70 reasons for acceptance and 38 reasons for rejection. The reasons were categorized into themes following thematic analysis and summarized, if shown similar (i.e., “good taste” and “I like the taste”) [34], whereas duplicates were excluded by the researcher. Following categorization, 10 reasons for acceptance (health, familiarity, good taste, positive sensory properties, appropriateness, special person, special occasions, good association with other food, culture, curiosity) and 6 reasons for rejection (danger, negative sensory properties, distaste, disgust, bad association with other foods, inappropriateness) resulted that were used in the main study. The obtained reasons were aligned with reasons that were found through reviewing literature about factors determining children’s food choices and preferences.

The databases Web of Science and PubMed were screened for relevant literature using the following key words: “children”, “food behavior”, “food choice”, “dietary choice”, “food acceptance”, “food rejection”, “food selection”, “food preferences”, “eating behavior”, “disliking”, “liking”, “reasons” and “factors”. The resulting literature was checked for relevance and references cited in each article, which were examined for further related studies. Only literature with full access and written in English were included, but no constraints were set in terms of date of publication. Studies that were considered eligible focused on factors and reasons in food choice in children and adults. 

A total of 52 articles were found to be eligible according to the above criteria, which were scanned for children’s reasons to accept or reject food (see Table S1). As some reasons from the literature search overlapped with reasons from the interviews, the most common reasons were selected by a researcher. The final stimuli are listed in the following section, “Questionnaire”.

### 2.4. Questionnaire

A questionnaire was developed to gain insight into reasons for accepting or rejecting food stimuli in children. These reasons were collected via a check-all-that-apply (CATA), which has been previously shown to be an easy, quick and child-friendly tool to collect spontaneous responses to food preferences in pre-adolescents [18,19,35]. A number of 10 reasons for each acceptance and rejection was aimed to be included in the final questionnaire, which is an appropriate number of items to use for a CATA questionnaire [36]. Reasons for acceptance included good taste, good smell, like of texture, like of appearance, healthy, familiar, special occasion, curious, culture, and parents; while reasons for rejection included bad taste, bad smell, dislike of texture, dislike of appearance, unhealthy, disgust, unfamiliar, bad experience, inedible, religion. Reasons were formulated as full sentences and formulated appropriately for children in this age group (pre-adolescents) to understand. As the literature on reasons for food acceptance and rejection was in English language, reasons were translated into Danish language by a native speaker. In case children had other or additional reasons for accepting or rejecting that were not stated, they were able to state these in an open-ended text box.

Additionally, familiarity of each stimulus was assessed by checking one of the response categories: I know it and tasted it before/I know it, but never tasted it before / I don’t know it. Children who tasted the food were asked to indicate their overall liking of the stimuli on a 7-point facial hedonic scale (response options: Super bad/Bad/Slightly bad/OK/Slightly good/Good/Super good) [37] and their willingness to re-taste it (yes/no). 

### 2.5. Study Procedure

At first, an instructor briefed the children that they were about to taste several food stimuli and that they would be asked about their self-reported reasons for accepting or rejecting these. It was emphasized that it was completely voluntary to participate. They were presented the questionnaire and asked to evaluate nine food stimuli by repeatedly going through the following steps: (a) tasting a sample (the children had the option not to taste the stimulus and had to note this accordingly); (b) giving reasons for accepting or rejecting it; (c) assessing the liking and willingness to re-taste, if tasted. The tasting sessions were conducted during school time at forenoon between 10:00–12:00 taking 45–60 min to complete. All necessary material was set up in the children’s habitual classrooms, where they normally eat. The children were allowed to sit at their ordinary seats and were not segregated by gender. The children were provided with a questionnaire, a plate, some forks and spoons, a napkin, a water cup for palate cleansing and a spitting cup. After tasting each food stimulus, children were asked to clean their palate with a water and dry bread crackers. The instructor guided the children through each stimulus assisted by two further assistants. The responsible teacher was present during the tasting session to keep the children as calm as possible. To minimize peer influence and stimulus boredom, two different serving orders were used (the other group received the same stimuli in the opposite serving order), so children sitting next to each other were served different foods [38,39].

### 2.6. Data Analysis

The CATA questionnaire was analyzed by calculating the frequencies of reported reasons for acceptance of all food stimuli. The same procedure was done for food rejection. Reasons that were checked by ≥50% of children were regarded as an important reason to choose the food stimuli. Gender differences were analyzed via Fisher’s exact test for all food stimuli comparing the counts of girls and boys for each reason. Additional reasons for acceptance and rejection stated through the open-ended text format were analyzed via thematic analysis, which is appropriate for analyzing qualitative data in this context [34].

For analyzing liking data of the 7-point facial hedonic scale, the mean liking (±SEM) was calculated. Gender differences were obtained comparing the means for liking via two-sample Student’s *t*-test. The willingness to re-taste was expressed as the proportions of girls and boys who were willing to re-taste the stimuli using Fisher’s exact test. The familiarity of the stimuli was expressed as the frequency of children who had tasted and not tasted the stimuli previously/who did not know the stimuli. Food stimuli were regarded as familiar if tasted previously by ≥50% of children. χ^2^-test was used to test if there was a difference in serving order of the stimuli between the two groups comparing the frequencies of the totals of acceptance and rejection of the stimuli from each serving group. The comparison was conducted for each stimulus and conducted separately for acceptance and rejection. The level of significance was set to *p* ≤ 0.05.

Statistical analyzes were conducted via XLSTAT (Addinsoft 2019; XLSTAT 2019.2.3; Boston, USA) and visualized via Microsoft^®^ Excel^®^ (for Office 365 MSO (16.9.11); Version 1902, Redmond, WA 98052 USA).

## 3. Results

In total, boys gave 2270 reasons and girls gave 1832 reasons for accepting foods, while boys gave 704 reasons and girls gave 585 reasons for rejecting foods. There were 13 cases across several food stimuli with no responses arising from children with allergy and/or intolerances, who could not taste specific foods and were therefore excluded from the analyzes. There were no differences when comparing children with the two different serving orders (*p* = 0.72), so in the following all data are merged together.

**Figure 1:**
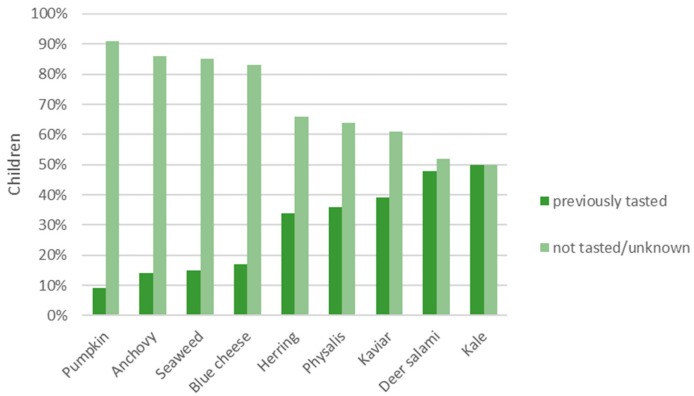
Familiarity of food stimuli from the least previously tasted and most unknown (left) to the most tasted food stimuli (right); *n* = 201–205 children.

Most stimuli were rather unfamiliar as most of the food stimuli had been previously tasted by <50% of the children (see Figure 1). Pumpkin, anchovy, seaweed and blue cheese were the least previously tasted and most unknown food stimuli, while herring, physalis and caviar were a bit more familiar to the children. The most previously tasted food stimuli were deer salami and kale which were familiar to approximately half of the children.

### 3.1. Children’s Reasons to Accept and Reject Foods

**Figure 2:**
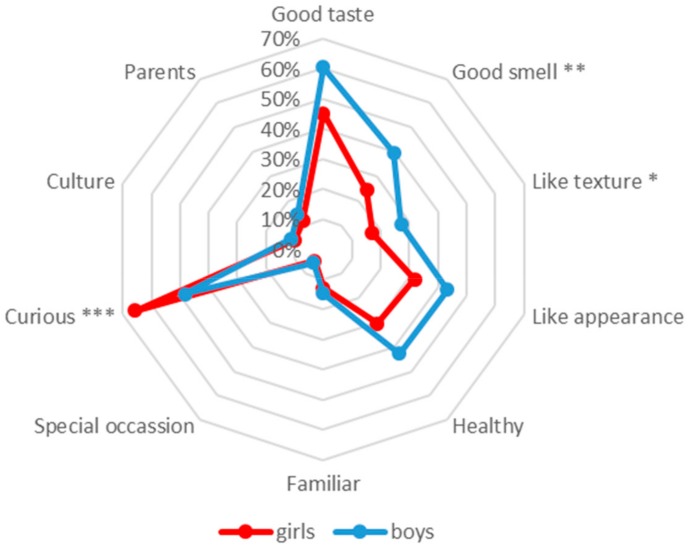
Reasons for accepting the food stimuli for girls and boys (pumpkin *n* = 79/80; kale *n* = 94/100; seaweed *n* = 92/89; physalis *n* = 93/94; caviar *n* = 86/80; herring *n* = 69/72; anchovy *n* = 53/66; blue cheese *n* = 69/62; deer salami *n* = 89/94); *n* = number of girls/boys accepting a food stimulus; the particular frequencies correspond to the lines below them and 0% corresponds to the center of the spider chart. Level of significance: * *p* ≤ 0.05, ** *p* ≤ 0.01, *** *p* ≤ 0.001.

Children’s reasons for acceptance are given in Figure 2. The reason of good taste (61%) was most important for boys and curiosity (66%) was most important for girls, which both were selected by ≥50% of children, respectively. The other reasons followed the same order of importance for both genders, which are listed from greatest to least (girls, boys): like of appearance (32%, 43%); healthy (30%, 43%); good smell (24%, 40%); like of texture (17%, 27%); familiar (13%, 15%); parents (12%, 15%); culture (10%, 11%); and special occasion (5%, 6%). As these reasons were stated by <50% of children they were therefore regarded as less important.

There were gender differences for several of the reasons given. Good smell (*p* ≤ 0.01) and like of texture (*p* ≤ 0.05) were more important in boys, whereas curiosity (*p* ≤ 0.001) was more important in girls for accepting the food stimuli.

**Figure 3:**
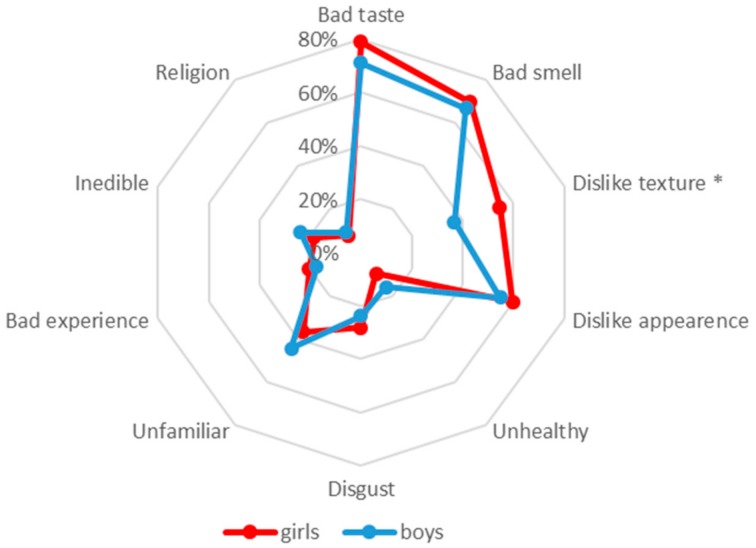
Reasons for rejecting the food stimuli for girls and boys (pumpkin *n* = 17/19; kale *n* = 4/4; seaweed *n* = 6/16; physalis *n* = 4/10; caviar *n* = 11/23; herring *n* = 29/30; anchovy *n* = 45/32; blue cheese *n* = 27/40; deer salami *n* = 9/10); *n* = number of girls/boys rejecting a food stimulus; the particular frequencies correspond to the lines below them and 0% corresponds to the center of the spider chart. Level of significance: * *p* ≤ 0.05.

Reasons for rejection are given in Figure 3. The reasons are listed from greatest to least (girls, boys) as follows: bad taste (79%, 71%); bad smell (70%, 67%); dislike of appearance (60%, 55%). These reasons were selected by >50% among both genders and dislike of texture (55%, 37%) was selected by the majority of girls. Significant gender differences were seen for dislike of texture (*p* < 0.05), which was more important in girls when rejecting the food stimuli.

### 3.2. Results from Open-End Response for Reasons to Accept or Reject Food

Children mentioned several other reasons for food acceptance and rejection. If children stated very similar reasons, these were merged into one category by the author. Other reasons for acceptance resulted in additional categories: grandparents, good association with other food, liking, challenge, good experience in childhood, ideals and price/value and other reasons for rejection resulted in processing of food, dislike and fear.

### 3.3. Liking and Willingness to Re-Taste

**Figure 4:**
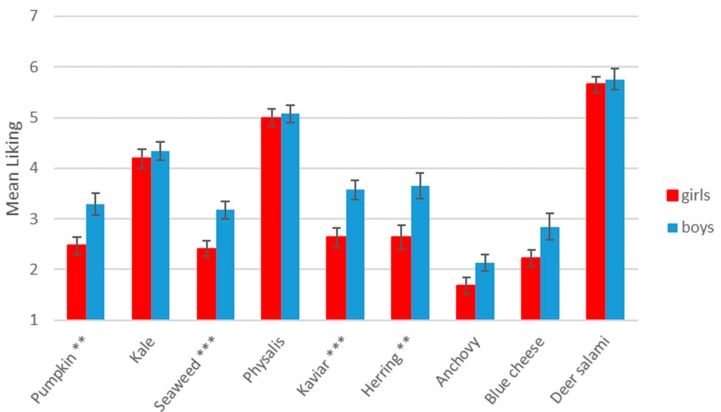
Mean liking (±SEM) of stimuli for boys and girls. Level of significance: ** *p* ≤ 0.01, *** *p* ≤ 0.001.

Food stimuli showed differences in mean liking, but differences between genders were shown (see Figure 4). In general, all food stimuli were more liked by boys, but this was only significant for pumpkin (*p* = 0.004), seaweed (*p* ≤ 0.001), caviar (*p* ≤ 0.001) and herring (*p* = 0.004).

**Figure 5:**
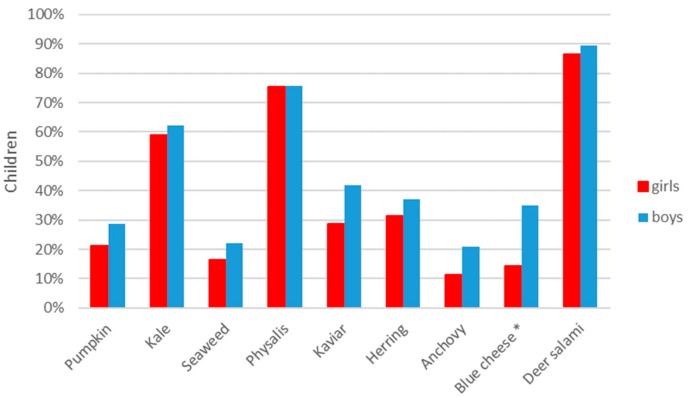
Children’s willingness to re-taste the food stimuli for boys and girls. Level of significance: * *p* ≤ 0.05.

Children’s willingness to re-taste the food stimuli was rather low, but it varied somewhat between stimuli (see Figure 5). In general, boys showed a higher willingness to re-taste the stimuli, but a significant gender difference was only shown for blue cheese (*p* ≤ 0.05).

## 4. Discussion

The present study investigated children’s self-reported reasons for accepting and rejecting specific food stimuli and if there were gender differences for these. We could only partly confirm that sensory attributes are children’s main drivers in food acceptance and rejection, as hypothesized, but we were able to show some gender differences in reasons for accepting and rejecting specific food stimuli, which confirmed our hypothesis. The results will be discussed more in detail in the following sections.

### 4.1. Children’s Reasons for Accepting Foods

Curiosity was the most important reason in girls (66%) and was shown to be significantly more important in girls than boys. This suggests that girls may be more influenced by curiosity than boys when deciding to try rather novel food or they may just be more aware of this influence than boys may be. Although, curiosity was selected by 48% of boys, it was the second most important reason and a key contributing factor. Interestingly, the study showed a very high importance for curiosity for the food stimuli that were mainly unfamiliar in the current study. It seems like the children were very interested to taste the novel foods, which could result from their natural drive to explore and discover [40,41,42]. Children’s curiosity emerges from birth and seems to decrease in adulthood [43]. In accordance with our study, it was previously shown that unfamiliar foods were accepted because of an interest evoked at the thought of consuming them [44], and that children’s food choice was influenced by a combination of curiosity and hesitancy when tasting new foods [45]. The fact that curiosity was so important may be used by researchers, schools and parents when trying to motivate children to try tasting novel foods [39,41,46]. Also, it remains to be determined what stimulates curiosity the most (e.g., the food itself, the eating situation or something else). A factor could be that the children regarded our tastings as a special event outside of the regular teaching class, which may have triggered the children to be more curious in trying the foods. However, studies examining children’s curiosity in connection to food choice are still rare but could be an interesting area to explore more in the future.

Good taste was the most frequently stated reason in boys (61%) and the second most frequently stated reason among girls (45%). We could not show a direct difference between genders, suggesting good taste is almost equally important in girls and boys. The high frequency of good taste is in accordance with a study on 6–14-year-old children, where 41%–59% of the reasons for liking specific foods resulted to be good taste [16]. Likewise, Fallon and co-workers suggested that good taste is one of the most influential factors in children’s and adult’s food acceptance [4,5,47].

Children selected appearance as the third most important reason (43% in boys and 32% in girls). The role of appearance in food choice has previously been demonstrated, where the serving style/shape of snack vegetables [48] and food plate presentation [49] clearly influenced children’s preferences for them. The literature on children’s preferred appearance of food is still relatively sparse, but it certainly seems to be an area worth further investigation. Interestingly, boys showed higher frequencies for all sensory properties, but these were only significant for good smell and liked texture. However, good smell and liked texture were selected by <50% of girls and boys and might therefore require more investigation to draw further conclusions.

Health and familiarity did not play a key role in this study, which aligns with previous findings, where familiarity was chosen less when choosing what to eat [50]. This contradicts research showing that familiarity influences children’s food choices and preferences to a considerable degree [51]. The fact that familiarity was primarily selected in connection with food rejection in this study, might be explained by the low familiarity of the food stimuli considered. A higher frequency for familiarity in boys (15%) than girls (6%) could be observed, which was also demonstrated previously, where familiar foods were more preferred by school-aged boys compared to girls [12]. Still, this study could not demonstrate a significant gender difference. Although environmental and social factors such as culture [52] and parents [53,54] are known to play great roles when choosing food, these reasons seemed less important for most children in accepting and rejecting food in the current study. Culture and parents may be more indirect influences and children may not pay attention to them directly. Additionally, by observing the children it was seen that—despite instructions not to interact—children challenged each other a few times to taste the foods (like the anchovy) they, e.g., felt disgusted by. Accordingly, some children—mainly boys—stated in the other reason category that they felt challenged to trying the foods.

### 4.2. Children’s Reasons for Rejecting Foods

In the present study, food rejection was shown to be more dominant in girls than in boys, which was also demonstrated previously, where women rejected more foods than men [25]. Women also appeared to be more sensitive to disgust than men, which might explain the slightly higher frequency of the reason disgust in girls (28%) than boys (24%) in the current study. The most important reasons for food rejection were within sensory attributes including bad taste, bad smell, disliking appearance and disliking texture, which were selected by >50% of both girls and boys. This highlights the role of sensory attributes in children’s food rejection. The reason of bad taste occurred most frequently, which is in accordance with Koivisto and Sjödén (1996), who investigated reasons for food rejection in children. The high relevance of the reasons of bad smell and dislike of appearance could be explained by the fact that they act as a “warning signal” to reject foods that are considered offensive or even disgusting because they could be potentially dangerous [47]. Texture is a strong influence in children [9,55] and the current study identified that dislike of texture was more important in girls compared to boys. Although it could not support a significant difference between genders for any other sensory properties [25,56], a recent study showed that females tended to give more responses for texture [57], which underpins the results of the current study.

The reasons of unfamiliarity, inedible, disgust, unhealthy, bad experience and religion were not very important compared to the sensory properties in the rejection of the food stimuli. However, unfamiliarity was demonstrated to be the second most important reason after the sensory attributes, which conforms with findings that taste and familiarity affect children’s food choices greatly [58]. Research has also established a connection between dislike and the rejection of unfamiliar food [14,59,60]. Unhealthy was the third least stated reason, which contrasts previously conducted research finding that health was one of the most important determinants in adult’s food choices [50]. Children are probably too young to understand what effects the consumption of food or its nutritional value may have [61].

The same seems to apply to bad consequences in children’s rejection of foods. In addition to that, many children did not know and had not tried the stimuli before, which could be an explanation as to why this reason was not relevant for the children. Inappropriateness and disgust were only of very minor importance, which contrasts former research stating that these are one of the main reasons for food rejection in children and adults [5,47]. An explanation could be that the selection of stimuli was considered “appropriate” to most of the children in the present study. Interestingly, disgust was mostly selected for blue cheese and foods from fish-origin (i.e., anchovy, caviar and herring) suggesting that children could be more disgust-sensitive towards these foods.

### 4.3. Liking and Willingness to Re-Taste Specific Foods

The current study showed that boys liked most of the stimuli more than girls, which aligns with previous research, where men tended to give more likes than dislikes compared to women and vice versa [62]. Furthermore, boys liked most of the fish products of this study (caviar and herring) more, which was also shown previously in children [12]. However, the current study could not confirm a significant higher preference for meat products, as opposed to Caine-Bish and Scheule (2009), who investigated gender differences in children’s food preferences [12].

The most liked food stimuli—like deer salami, physalis and kale—were also demonstrated to be the food stimuli the children would like to re-taste the most. This can be explained by the theory that positive food experiences can elevate its liking [63]. The remaining stimuli showed a rather low inclination to be re-tasted, which is attended by the low means of liking.

There was no significant gender difference in regard to re-tasting the food products, except for blue cheese, which boys were more willing to re-taste. These results propose that both genders may be similar in terms of re-tasting foods. Since boys generally liked the foods more than girls, it is remarkable that their absolute level of willingness to re-taste was almost the same. 

### 4.4. Strengths and Limitations

The food stimuli can be explained as rather unfamiliar to the children. Still, the number of accepted stimuli was very high, which contrasts research showing that familiar foods are more likely to be accepted compared to unfamiliar foods [44]. One explanation for this phenomenon could be that children often seem to eat more foods away from home, which they would refuse at home [64]. Food choices are often influenced by the social and physical environment and its various contexts [65]. This observation could explain the high percentage of accepted stimuli in this study.

Additionally, the food stimuli covered several of foods groups including vegetables, fruits, fish and meat-based products and dairy. However, the food stimuli might be quite particular and therefore not reflect an entire food category, especially considering that both the products within a food category and their preparation methods can vary a lot. Therefore, the results of the current study may be unique to these foods as opposed to these types of foods or foods in general. Consequently, future research should focus on a broader range of stimuli to be able to generalize. The preparation of the stimuli (pre-cutting into bite-sized pieces) may have increased the number of accepted stimuli, as it was shown in a previous research [48,66]. Hence, this knowledge can be used to make food more appealing for children by using appropriate serving styles, i.e., schools should pre-cut fruits and vegetables.

Although it was a strength that the schools were located in various areas of Copenhagen, which represented children from different socio-economic backgrounds, the study might be limited to children living in the city as food choices of children from the city and rural areas can vary [52]. Additionally, age can be a factor in children’s food choices [27], but in the current study the age investigated was limited to 10–13-year-old children and was not discussed due to the small difference in age.

### 4.5. Implications for Health Behavior

Previous studies showed that unfamiliar foods are often rejected [22,24], which was however here not the case; it was shown that unfamiliar foods had a rather high acceptance. The fact that our research team came from outside the school could have provoked a natural interest in the children to try novel foods. Therefore, it could be relevant to have more projects in schools, where children are exposed to new and healthy foods. To be effective, teachers should be advised to implement health professionals or organizations disseminating taste and healthy foods in their curriculum. When children are exposed to food, this should be conducted as interestingly as possible by, e.g., creating tasting experiments and games with food, challenging the children to taste, letting them explore and teaching them about interesting food facts [46]. The fact that curiosity seems promising may be integrated better into future interventions.

Additionally, the study provides support for the importance of sensory properties of foods in both acceptance and rejection. Previously conducted sensory education programs have shown positive effects on encouraging children to try novel foods and reducing food neophobia [40,67]. The sensory lessons can be used to make children well-informed consumers through activities that focus on their use of senses when tasting food and appeal to their interest and curiosity. Additionally, the knowledge about gender differences in reasons for accepting and rejecting food can be used, e.g., in school cantinas, to make healthy foods more interesting, adjusted for both girls and boys in terms of appearance, smell, taste and texture. A better understanding of children’s reasons for accepting and rejecting foods will provide parents and practitioners alike with knowledge about how to best support the development of healthy eating behavior without disregarding the role of gender in children’s food preferences.

In this respect, our findings provide suggestions for practical implications and a scope for further research on sensory education and food exposure programs, which have potential to motivate children to try new foods: children’s curiosity seems to be the optimal precondition to taste foods they have never tasted before. Exposure to novel healthy food products can help to increase children’s food acceptance and thereby broaden the variety of their diet [40]. To promote dietary variety from very early on is essential as food preferences formed in early childhood do show some tracking into adulthood and may contribute to build the basis for food habits later in life [68]. Poor dietary choices in adulthood can lead to the development of chronic diseases like heart disease, stroke, cancer, chronic respiratory diseases and diabetes, which are the major causes of mortality worldwide [69]. Consequently, as a varied diet including healthy foods is assumed to be beneficial for health and part of a healthy lifestyle, such approaches could contribute to healthier children [70]. 

## 5. Conclusions

The results suggest promising insights into children’s reasons to accept or reject foods and that gender differences should be considered when investigating children’s food choices and preferences. More focus should be on the role of sensory attributes in food acceptance and rejection and the importance of children’s curiosity as a driver to accept novel foods. This knowledge can be used to increase the dietary variety of healthy foods.

## Supplementary Materials

The following are available online at https://www.mdpi.com/2072-6643/11/10/2455/s1, Table S1: List of references and reasons in food choice and food preferences in children and adults.
