# Supplementary material for: Children’s Self-Reported Reasons for Accepting and Rejecting Foods
Source: Nutrients. 2019 Oct 14;11(10):2455. doi: 10.3390/nu11102455 (PMC6836127; doi:10.3390/nu11102455)
Supplement: Supplementary file 1 [file nutrients-11-02455-s001.pdf]

**Supplementary Materials:**

**Table S1.** List of references and reasons in food choice and food preferences in children and adults.

| References | Reason |
|---|---|
| 1. Ahrens 2015 | taste, culture |
| 2. Baxter et al. 2000 | texture, appearance |
| 3. Brown & Harris 2012 | disgust |
| 4. Caine-Bish & Scheule 2009 | familiarity |
| 5. Clark 1998 | taste, flavor |
| 6. Contento et al. 1993 | parents |
| 7. De Moura 2007 | disgust, familiarity |
| 8. Dovey et al. 2008 | familiarity |
| 9. Drewnowski et al. 2012 | sensory properties, familiarity |
| 10. Fallon & Rozin 1983 | taste, appropriateness, disgust |
| 11. Finistrella et al. 2012 | familiarity |
| 12. Fisher et al. 2002 | parents |
| 13. Flight et al. 2003 | culture |
| 14. Galloway et al., 2005 | parents |
| 15. Gibson et al., 1998 | liking, health |
| 16. Heidelberger & Smith 2014 | health |
| 17. Hetherington 1996 | taste, appearance |
| 18. Hughner & Maher 2006 | parents |
| 19. Koivisto & Sjödén 1996 | taste |
| 20. Koivisto & Sjödén 2012 | health, taste, familiarity, eaten by self and others, contents, economy, smell/appearance |
| 21. Kourouniotis et al., 2016 | taste |
| 22. Kühn & Thybo 2001 | texture |
| 23. Luckett & Seo 2015 | texture |
| 24. Maga 1974 | taste |
| 25. Martins & Pliner 2005 | sensory properties, interest of consuming food |
| 26. Murimi et al. 2016 | sensory properties, taste, appearance |
| 27. Mustonen & Tourila 2010 | sensory properties |
| 28. Nago et al., 2012 | sensory properties, food safety |
| 29. Nederkoorn et al. 2015 | texture |
| 30. Nehring et al. 2015 | taste |
| 31. Nicklaus 2016 | sensory properties, parents |
| 32. Nicklaus & Issanchou 2007 | culture, sensory properties, social influences |
| 33. Nicklaus et al. 2004 | sensory properties, taste |
| 34. Oellingrath et al., 2013 | parents, health |
| 35. Olsen et al. 2012 | appearance |
| 36. Park & Cho 2016 | taste |
| 37. Pliner 1982 | familiarity |
| 38. Pollard et al. 1998 | health |
| 39. Pollard et al. 2002 | appearance, texture, culture |
| 40. Rozin & Vollmecke 1986 | appropriateness, disgust, health |
| 41. Savage et al. 2007 | parents |
| 42. Scaglioni et al. 2011 | parents |
| 43. Steptoe et al. 1995 | familiarity |
| 44. Stevenson et al. 2007 | sensory properties |

| | |
|---|---|
| 45. Szczesniak & Kahn 1971 | texture |
| 46. Taylor et al. 2015 | familiarity |
| 47. Thomson 1989 | culture |
| 48. Thybo et al. 2004 | texture |
| 49. Van der Horst 2012 | parents |
| 50. Werthmann et al. 2015 | sensory properties, taste, appearance |
| 51. Zajonc 1968 | familiarity |
